# Update on resistance to thyroid hormone syndromeβ

**DOI:** 10.1186/s13052-020-00929-x

**Published:** 2020-11-11

**Authors:** Hongping Sun, Lin Cao, Rendong Zheng, Shaofeng Xie, Chao Liu

**Affiliations:** 1 Endocrine and Diabetes Center, Affiliated Hospital of Integrated Traditional Chinese and Western Medicine, Nanjing University of Chinese Medicine, No.100, Shizi Street, Hongshan Road, Nanjing, 210028 China; 2 Jiangsu Province Academy of Traditional Chinese Medicine, No.100, Shizi Street, Hongshan Road, Nanjing, 210028 China

**Keywords:** Thyroid hormone resistance, Thyroid hormone receptor β, Thyroid hormone, Gene mutation, Autosomal genetic disease

## Abstract

Resistance to thyroid hormone syndrome (RTH) is an autosomal dominant or recessive genetic disease caused by mutation of either the thyroid hormone receptorβ (THR-β) gene or the thyroid hormone receptorα (THR-α) gene. RTH due to mutations of the THR-β gene (hereafter, RTH-β) is characterized by a decreased response of the target tissue to thyroid hormone, increased serum levels of free triiodothyronine (FT3) and/or free thyroxine (FT4), and inappropriate secretion of thyroid-stimulating hormone (TSH, normal or elevated). Clinical manifestations of RTH-β vary from hyperthyroidism to hypothyroidism or simple goiter, and RTH-β is often misdiagnosed clinically. The present review was prepared for the purpose of expanding knowledge of RTH-β in order to reduce the rate of misdiagnosis.

## Introduction

Resistance to thyroid hormone syndrome (RTH) is an autosomal dominant or recessive genetic disease caused by mutation of either the thyroid hormone receptorβ (THR-β) gene or the thyroid hormone receptorα (THR-α) gene. RTH due to mutations of the THR-β gene (hereafter, RTH-β) is characterized by a decreased response of the target tissue to thyroid hormone, increased serum levels of free triiodothyronine (FT3) and/or free thyroxine (FT4), and inappropriate secretion of thyroid-stimulating hormone (TSH, normal or elevated). Clinical manifestations of RTH-β vary from hyperthyroidism to hypothyroidism or simple goiter, and RTH-β is often misdiagnosed clinically. The present review was prepared for the purpose of expanding knowledge of RTH-β in order to reduce the rate of misdiagnosis.

Thyroid hormone (TH) acts via nuclear thyroid hormone receptors (TRs), and several TR isoforms (e.g., TR-α1, TR-α2, TR-β1, TR-β2) are encoded by distinct genes, *THR-α* and *THR-β*, and show differing tissue distributions. Resistance to TH syndrome (RTH) was first reported by Refetoff et al. [1] in 1967and is an autosomal dominant or recessive genetic disease caused by mutation of either *TR-α* or *TR-β* [2]. The main characteristic of RTH is insensitivity to TH, with elevated serum levels of free thyroxine (FT4) and free triiodothyronine (FT3) accompanied by a normal or slightly elevated serum thyroid stimulating hormone (TSH) level. Currently, RTH is defined by impaired sensitivity to TH [3–5]. In view of distinct phenotypes associated with mutation of the different genes, we propose the use of the terms ‘RTH-α’ and ‘RTH-β’ to denote the gene mutation responsible in cases of RTH. This will help to clarify discussions of the clinical phenotypes of RTH as well as allow the naming of RTH phenotypes associated with new gene defects [4].

The severity of the clinical phenotype of RTH-α seems to depend on the location and type of mutation in *THR-α*. The most frequent manifestations of RTH-α include anemia, constipation, and growth and developmental delay. In addition, serum free triiodothyronine (FT3) levels can be high-normal to high, free thyroxine (FT4) and reverse T3 (rT3) levels can be normal to low, and the serum thyroid-stimulating hormone (TSH) level is normal or mildly raised. RTH-α cases, particularly those with mild mutations or those diagnosed in early life, may response well to LT4 treatment [6]. Studies published in 2012 indicate that mutant TR-α1 is associated with abnormal levels of TH but normal levels of thyrotropin as well as growth retardation and mildly delayed motor and cognitive development [7, 8]. RTH-α is a rare disease that can be described simply as above.

RTH-β is a dominant or recessive disorder that occurs in familial and sporadic cases with an incidence of about 1:40,000–1:50,000, and about 85% of RTH-β cases have a *THR*-*β* gene mutation [3, 9, 10]. Herein we review the recent literature to provide an update on the pathogenesis, clinical presentation, and treatment of RTH-β.

### Classification

RTH-β is generally divided into three categories: general RTH-β, pituitary RTH-β, and peripheral RTH-β [11]. Patients with general RTH-β usually have normal thyroid function and normal skeletal development but may have a low intelligence quotient. Normal thyroid function in these patients is achieved by compensation with increased TH production. Patients with pituitary RTH-β usually have symptoms of hyperthyroidism, with inappropriate secretion of TSH and elevated T4 and T3. Patients with peripheral RTH-β commonly have symptoms of hypothyroidism, except that the TSH level is normal, and the peripheral tissue is not sensitive to TH compared with the pituitary gland.

### Pathogenesis

RTH is mainly caused by defects in TR, which is encoded by the *THR-α* and *THR-β* genes of chromosome 17 and chromosome 3, respectively. The molecular structures and sequences of the TR-α and TR-β proteins are very similar, with four isomers denoted as TR-α1, −α2, −β1 and -β2, all of which can bind with TH except for TR-α2. These receptor isoforms have distinct distributions. TR-α1 is widely distributed, particularly in the heart and muscle tissue, while TR-α2 is widely distributed but unable to bind hormones. TR-β1 is mainly distributed in the brain, liver and kidney, whereas TR-β2 is mainly expressed in the hypothalamus and pituitary gland [12, 13].

Most cases of RTH-β are caused by a point mutation in TR-β isoforms, but the exact molecular mechanism remains unclear. The most common form of RTH-β is caused by a small defect in one arm of a TR-β gene, while RTH-β doesn’t appear with an individual missing a TR-β arm. These findings suggest that RTH-β is not simply due to a decrease in functional TRs, possibly because the interaction between the mutant TR-β and the wild type TR-β.

Most mutations of the TR-β are located in the two “hot spots”, separated by a highly conserved region of 80 amino acids known as the “cold zone” from codons349 to 429. Recently, a new mutation point has been discovered, and the range of hot spots has been extended to codons 309 to 353 and codons 374 to 461. In addition, a third mutation cluster has been confirmed with the range of codons 234 to 282 [14]. *THR-β* is located on chromosome 17 and consists of 10 exons. More than 100 mutations have been reported; with the exception of one family, all mutations are located on exons 7–10 [15, 16].

About 140 TR-β mutations have been linked to RTH-β to date [17]. Kurozumi et al. reported a RTH-β case with the P453A mutation of TR-β in which the patient showed persistent tachycardia without a family history of RTH-β [17]. The patient’s symptoms were relieved upon treatment with beta blockers, and the authors noted that the treatment for RTH-β differs from that for Graves disease or a pituitary TSH tumor. Genetic testing should be considered first even if a patient has no known family history of RTH-β, if inappropriate TSH secretion is observed and a TSH tumor must be ruled out [17].

Although in most cases, RTH-β is an autosomal dominant disorder, it can also be an autosomal recessive disorder [18]. Takeda et al. [19] reported the molecular basis of generalized RTH-β in a consanguineous family unique for its autosomal recessive mode of inheritance.

### Clinical manifestations

The clinical manifestations of RTH-β, and the severity of the disease vary greatly. About 65–95% of the RTH-β patients have goiter, and other symptoms include sinus tachycardia, abnormal psychology, short stature, development retardation, growth retardation, hair loss, and repeated infection of ear, nose and throat [20]. Agrawal et al. [18] reported cases of familial RTH-β in which the proband and his mother both had symptoms of hyperthyroidism with A317T mutation, such as palpitations and sweating, and the diagnosis was considered pituitary RTH, prompting treatment with beta blockers. Diverse mutations have been shown to result in different clinical manifestations.

Often no obvious clinical manifestations appear for general RTH-β. However, serious clinical symptoms can appear, and some patients show relatively light or moderate clinical signs in some general RTH-β cases. Mild to moderate hypothyroidism may appear in pituitary RTH-β, usually without pretibial mucous edema and proptosis. Peripheral RTH-β is rare, and such cases mainly show signs and symptoms of hypothyroidism. The clinical signs of RTH-β usually include goiter with normal thyroid function or hypothyroidism, and the TSH level is normal or elevated [18].

### Work-ups

If tests for thyroid function show elevated FT3 and FT4 levels with normal or elevated TSH and normal thyrotrophin receptor antibody (TRAb) levels, RTH-β should be suspected. The thyrotropin-releasing hormone stimulating test indicates that TSH has good reactivity to thyrotropin-releasing hormone stimulation. Genetic testing is appropriate to identify mutations in *THR-β.* Other examinations such as imaging studies can show the absence of a lesion in the pituitary gland in RTH-β.

### Diagnosis and differential diagnosis

A diagnosis of RTH-β should be confirmed according to genetic family history, age at onset, goiter and other clinical manifestations, combined with hyperthyroidism, normal or elevated TSH, and TR-β genetic testing. Related evaluations should be completed to rule out other diseases that cause elevated TSH, such as pituitary adenoma, primary hypothyroidism, resistance to TSH receptor syndrome due to a TSH receptor mutation, drug-induced elevated TSH, ectopic TSH syndrome, and others [21].

Anti-thyroid drugs should be discontinued for 1 month and levothyroxine (L-T4) discontinued for about 6 weeks for suspected cases of RTH-β. Cases of pituitary TSH tumor also show elevated TH and unsuppressed TSH levels, and relevant examinations need to be completed for differential diagnosis, such as pituitary magnetic resonance imaging (MRI) and a thyrotropin-releasing hormone–stimulating experiment [22]. Genetic diagnosis is the gold standard for RTH-β currently. Although patients and their relatives all should undergo genetic examination, about 15% of RTH patients will have no genetic abnormality [23]. A levotriiodothyronine (L-T3) inhibition test is also recommended in cases of suspected RTH-β for classification and treatment, but this technique is not widely developed in China. Moreover, L-T3 is expensive and can have harmful effects on cardiac function [24].

### Treatment

Most RTH-β patients who do not have clinical symptoms donot need treatment, because increased TH production can compensate for resistance of tissues. Selective beta-blockers can be considered for patients with obvious tachycardia during resting. Because atenolol cannot inhibit the conversion of T4 to T3, it is the best choice for RTH-β with tachycardia. Triiodothyroaceticacid (TRIAC) or dextrothyroxine (DT4) also can be chosen to treat thyrotoxicosis. Patients with hypothyroidism can be treated with L-T4, but the required dose is often high. Reduction of the elevated TH level is not recommended, because such treatment could lead to signs and symptoms of low metabolism, especially for newborns with in the first month after delivery when TH is essential in nervous system development [14]. In addition, elevated TSH levels caused by anti-thyroid drugs may lead to goiter and pituitary hyperplasia [14]. Maruo et al. [16] reported a case of RTH-β in which the patient showed signs and symptoms of mental retardation, hyperactivity, insomnia, and low resting energy expenditure while receiving treatment with L-T4, and these signs and symptoms were alleviated after adjustment to a high dose of L-T3, including an increase in resting energy expenditure without the occurrence of thyrotoxicosis. Therefore, Maruo et al. suggested that L-T3 can be used as an effective treatment for severe RTH-β.

Lai et al. [25] reported a case of familial RTH-β with a mutation of A234T and discussed an effective treatment strategy. It is believed that anti-thyroid drugs can effectively relieve the thyrotoxicosis of RTH-β but may increase the risk of thyroid cell proliferation. The dopamine agonist bromocriptine can inhibit the inappropriate secretion of TSH, but the safety of bromocriptine and D-T4 remains controversial. TRIAC is currently the most promising drug for reducing both TH and TSH levels. In addition, L-T3 or L-T4 can be administered to patients with diffuse goiter or hypothyroidism. Overall, treatment of RTH remains challenging, and individualized treatment should be formulated according to the patient’s clinical manifestations.

### Reported associated conditions

#### RTH-β accompanied by ectopic thyroid gland

Guoetal [26]reported a case of rare RTH-β with an ectopic thyroid gland in which a 10-year-old girl was diagnosed with congenital hypothyroidism, treated with L-T4. Her TSH was not suppressed, and FT3 and FT4 levels were elevated after 10 years of follow-up without any symptoms of hypothyroidism and hyperthyroidism. Here physical development and cognition were normal. No pituitary tumor was found on MRI, and a large thyroid gland located in the tongue was revealed by ultrasonography. Octreotide inhibition test showed that TSH was decreased by 41.98%, and no gene mutations were found in THR-β, THR-α, TSHR, orguanine nucleotide-binding αsubunits. Guo et al. suggested that levothyroxine can be considered for RTH-β patients who show symptoms of hypothyroidism. For this case, the unusually elevated TSH may lead to thyroid hyperplasia in the tongue, for which treatment is essential. The dopamine agonist bromocriptine can inhibit inappropriate secretion of TSH and can be used in combination with 3,5,3′-triiodothyroacetic acid.

### 7.2 RTH-β accompanied by cardiomyopathy and diabetes mellitus

Wakasaki et al. [27] reported a case of a 60-year-old man with RTH-β complicated by cardiomyopathy, diabetes, and elevated TSH, and genetic testing revealed the A268D missense mutation in TR-β. The authors suggested that patients with RTH should undergo evaluation of heart function and glucose metabolism.

#### RTH-β accompanied by a pituitary TSH tumor

Teng et al. [28] reported one case of a 12-year old girl diagnosed with a pituitary TSH tumor and RTH-β. Resection of the sphenoid sinus pituitary adenoma was performed, and histological evaluation showed positive staining of TSH-producingβ cells, human chorionic gonadotropin-producingα cells, growth hormone-producing cells, prolactin-producing cells, and serum corticotrophin-releasing cells. Genetic testing for TR-β mutation showed the P453T mutation. So she was diagnosed with RTH-β accompanied by a pituitary TSH tumor. Thyroid function was improved significantly after the operation, and menarche came along with an increase in the patient’s height.

#### RTH-β accompanied by graves disease

Anti-thyroid drugs can keep the disease stable for patients diagnosed with RTH-β combined with Graves disease, and radioactive iodine and surgical treatment are not recommended because they may lead to pituitary hyperplasia. Sun et al. [29] and Sato et al. [30] reported cases of patients with RTH-β complicated by Graves disease that remained in stable condition by taking methimazole. Sivakumar et al. [31] reported a patient with RTH-β with hyperthyroidism, in whom hypothyroidism appeared after iodine 131 radioactive treatment. The patient needed a large dose of levothyroxine to maintain stable thyroid function.

#### RTH-β accompanied by papillary thyroid carcinoma

Karakose et al. [32] reported two cases of RTH-β combined with papillary thyroid carcinoma that were stable after surgical treatment and replacement therapy. In both cases, the required does of L-T4 was very high, and suppression of TSH to within the goal range was difficult to achieve. Also, symptoms of hyperthyroidism may appear in some patients, and then T3 or TRIAC could be considered. There is no consensus regarding the appropriate intervention for patients with RTH-β and thyroid cancer.

Aoyama et al. [33] reported a case of papillary thyroid carcinoma in which TSH levels could not be suppressed postoperatively, and thus, RTH-β was suspected. Genetic testing revealed theP453S mutationin TR-β, and the patient was diagnosed with RTH-β and papillary thyroid carcinoma. When the L-T4 dose was gradually increased to 350 μg/day, the TSH level was reduced and the patient didnot complain of any thyrotoxicosis. No recurrence of thyroid cancer was observed. Because up to 95% of RTH-β patients will experience diffuse goiter, long-term stimulation of the thyroid gland by elevated TSH may be related to the formation and growth of thyroid carcinoma or thyroid nodule, but further research is needed to confirm this possibility.

## Conclusion

RTH-β is an autosomal dominant or recessive inherited disease with different clinical manifestations, and most cases are caused by TR-β mutation and generally do not require treatment. In cases with obvious thyrotoxicosis, TRIAC is effective, and beta-blocker treatment may be considered for patients with obvious tachycardia. For patients with hypothyroidism, L-T3 or L-T4 can be considered (Table 1). There are no guidelines or expert consensus on the treatment of RTH accompanied by other diseases, and thus, further research is needed. The syndrome should be suspected in patients with increased serum TH level, accompanied by a normal or elevated TSH concentration. The affected patients require individualised management [34].

**Table 1:** Key points of differentiation between RTH-α and RTH-β

TR types	RTH type	Mutation receptor	Clincal manifestations	Treatment
TR-α1: Heart, muscle tissue TR-α2: Widely distributed TR-β1: Brain, liver, kidney TR-β2: Hypothalamus, pituitary gland	RTH-α	TR-α	Anemia, Constipation, Growth retardation, Developmental delay, Motor and Cognitive development delay	L-T4
	RTH-β	TR-β	General, Pituitary and Peripheral RTH-β Vary from hyperthyroidism to hypothyroidism or simple goiter	L-T4 or L-T3, TRIAC, DT4, beta-blockers

## Data Availability

Not applicable.
